# Risk factors associated with HIV infection in four large Brazilian blood centers: A multicentric case-control study (2009–2017)

**DOI:** 10.1016/j.htct.2025.103863

**Published:** 2025-07-05

**Authors:** Fernanda Dominique de Souza Gonçalves, Flávia da Costa Silva, Isabel Cristina Gomes Moura, Cesar de Almeida-Neto, Brian Custer, Ester Cerdeira Sabino, Paula Loureiro, Carolina Miranda, Luiz de Melo Amorim Filho, Tassila Salomon

**Affiliations:** a Faculdade Ciências Médicas de Minas Gerais (FCM-MG), Belo Horizonte, MG, Brazil; b Universidade Federal de Minas Gerais (UFMG), Belo Horizonte, MG, Brazil; c Fundação Pró-Sangue Hemocentro de São Paulo (FPS), São Paulo, SP, Brazil; d Vitalant Reserach Institute, San Francisco, CA, USA; e University of California San Francisco, San Francisco, CA, USA; f Instituto de Medicina Tropical da Universidade de São Paulo (IMT, USP), São Paulo, SP, Brazil; g Fundação de Hematologia e Hemoterapia de Pernambuco (Hemope), Recife, PE, Brazil; h Fundação de Hematologia e Hemoterapia de Minas Gerais (Hemominas), Belo Horizonte, MG, Brazil; i Instituto Estadual de Hematologia Arthur de Siqueira Cavalcanti (Hemorio), Rio de Janeiro, RJ, Brazil

**Keywords:** HIV Risk factors Blood donors

## Abstract

**Background:**

Strategies to reduce contamination by transfusion-transmissible infections are constantly evolving. Over the years, HIV residual risk has decreased in several countries. However, in Brazil a recent study showed that the residual risk remains substantially higher than in other countries. Continuous surveillance of risk behaviors for infection in donors can help in pre-donation screening to reduce the risk of HIV in blood transfusions.

**Methods:**

This analysis evaluated risk factors related to HIV infection among blood donors from four large Brazilian blood centers located in São Paulo, Rio de Janeiro, Belo Horizonte and Recife, from 2009–2017. A binary logistic model was used to evaluate any association between risk characteristics and behaviors and the occurrence of HIV. The significant variables were included in a saturated model, to which the backward strategy was applied to arrive at the final model. The analyses were carried out using the R program version 4.1.2 and p-value <0.05 was considered significant.

**Results:**

A total of 1507 blood donors were included in the study, 716 were HIV positive and 791 were uninfected controls. Demographics significantly associated with infection were: Male sex, incomplete secondary education, separated/divorced/widowed, and bisexual/homosexual orientation. Behaviors most strongly associated with infection were: workplace exposure, intravenous drugs and men who had sex with other men.

**Conclusion:**

The risk factors identified suggest that the blood donor screening process in Brazilian blood centers does not adequately identify donors at increased risk for HIV and further studies should be carried out to support changes to improve the process.

## Introduction

Blood transfusions are critical interventions widely used to treat a range of clinical indications.^1^ Strategies to reduce contamination by transfusion-transmissible infections (TTIs), such as clinical and epidemiological screening of blood donors, serological screening tests and microbiological inactivation are constantly evolving.^2^ Currently, the improvement in process controls of blood banks and the use of techniques, such as the nucleic acid amplification test (NAT) to detect the genetic material of HIV, have reduced the risks of TTIs.^3^

The risk of HIV transmission through transfusion following the adoption of safety interventions is called residual risk (RR). RR has decreased in countries that have adopted measures to control blood quality from the emergence of HIV to the present day. However, a recent study showed that in Brazil there is no evidence that there has been a decrease in RR over the last ten years. The RR of HIV transmission in Brazil is 5.46 per million transfused red blood cell units,^4^ remaining substantially higher than in other countries such as Germany (0.52 per million transfusions),^5^ France (0.40)^6^ and Canada (0.04).^7^ HIV contamination through transfusion is still possible even with current procedures due to factors such as the coincidence that the donation period is within the HIV immunological window.^4^ Furthermore, risks change over time and a study from Brazil demonstrated that donors using antiretroviral prophylaxis have different periods of the immunological window, and that the use of antiviral prophylaxis by blood donors in the acute phase of HIV infection delays seroconversion.^8^

In Brazil, the RR of TTI transmission decreased significantly after the implementation of NAT; however, it remained stable after this initial reduction and remains a major concern,^9^, ^10^, ^11^, ^12^ especially with the introduction of antiretroviral therapies, which prolong the immunological window. Cases of blood donors using antiretrovirals have already been reported in Brazilian studies, highlighting the need for greater surveillance in this context.^13^ Thus, transfusion safety measures need to be improved to reduce the RR of HIV transmission. In addition to the laboratory measures applied for quality control, the steps prior to blood collection must be assessed, which involves the process and evaluation of the donor during screening prior to donation.^14^ The identification of risk factors related to HIV infection, such as age, gender, sociodemographic factors, medical history and sexual behavior in pre-donation interviews can inform changes in the donor selection process during the eligibility interview.

Some studies have shown that Audio Computer-Assisted Structured-Interviews (ACASI) allow donors to report stigmatizing behaviors (such as sexual behaviors), and may help to close a gap in the pre-donation screening process.^15^^,^^16^ In addition, studies show that changing the approach to assessing infection risks can help in pre-donation screening to reduce the RR of HIV transmission.^15^, ^16^, ^17^, ^18^

This analysis evaluates risk factors related to HIV infection in blood donors from four large Brazilian blood centers from 2009–2017. This period of time precedes the federally mandated removal of sexual orientation as part of the donor selection process. Before 2020, Brazilian regulations did not allow donations from men who have sex with men (MSM) in the 12 months before donation. In May 2020, the Brazilian Supreme Court prohibited asking questions about sexual orientation to qualify blood donation candidates based on equal treatment of all potential blood donors.^19^

## Study design and methods

This study was part of the NHLBI REDS-II and III International program (Brazil). The study included Fundação Pró-Sangue in São Paulo, Fundação Hemominas in Belo Horizonte and Fundação Hemorio in Rio de Janeiro, all in the southeastern and most populous region of Brazil, and Fundação Hemope in Recife in Northeastern Brazil. Blood samples from each donation were screened at each center with two different enzyme immunoassay assays (EIAs) (third and fourth generation) performed in parallel. By standard procedure, if the EIAs were reactive or discordant, a Western blot was performed. After 2012, with the gradual implementation of NAT in Brazilian blood banks, a serological test was performed, carried out in duplicate. Risk factor interviews for HIV infection of donors were conducted from April 2009 to March 2017. In the first phase of the study, from April 2009 to March 2011, HIV positive (cases) and HIV negative (controls) donors were interviewed. In the second phase, from April 2011 to March 2017, only cases were interviewed. The two datasets were combined for this analysis. All subjects included in the study completed the risk factor questionnaire at the time of return to the blood center for notification and counseling. Potential controls were recruited into this study after donation. Controls tested negative for all infections for which donations are screened in Brazil and were randomly selected based on scheduled donation visits during the first phase.

The ACASI risk factor questionnaire was based on HIV risk interviews developed by the US Centers for Disease Control (CDC)^20^ but was modified to reflect potential risk behaviors in Brazil. The questionnaire included sociodemographic factors, incentives and motivations to donate, sexual history, and risks of sexual partners. A social matrix was used to obtain detailed information about sexual behaviors and other risks of the participant's last five sexual partners before blood donation. Other factors, including alcohol and drug use, medical history, other potential risk factors (like tattooing, piercing, acupuncture, grooming at barbershops or beauty salons) and workplace exposure, were also assessed. In the analysis, the sexual orientation of the donor was included, in addition to the self-reported behaviors reported during the interview. For example, donors who reported sex exclusively with same sex partners were classified as homosexual, if they also reported sex with opposite sex partners, they were classified as bisexual, and if they reported only opposite sex partners, as heterosexual.

## Statistical analysis

Summary descriptive statistics comparing cases and controls were generated and are presented as frequencies. Bivariable logistic regression was used to assess the unadjusted association between donor characteristics or behaviors and the occurrence of HIV. Significant variables from the bivariable models were included in a saturated model, to which a backward elimination strategy was applied to arrive at the final multivariable model. Results are presented as odds ratios (OR) and 95 % confidence intervals (95 % CI). The goodness of fit was evaluated by the Hosmer-Lemeshow test, and multicollinearity was evaluated by the variance inflation factor (VIF) statistic. The analyses were performed using R version 4.1.2 and a p-value <0.05 was considered significant.

### Ethical considerations

The original ACASI interview studies were reviewed and approved by Ethics Committees in Brazil and Institutional Review Boards (IRBs) in the United States. The study protocols were approved by the Federal Committee on Human Subjects (CONEP) of the Ministry of Health in Brazil as part of the REDS-II/III International Program, ethics committees at all participant blood centers, and also IRBs in the United States. This analysis of anonymized previously collected data was not reviewed by Ethics Committees or IRBs.

## Results

A total of 1507 blood donors participated in the study, of which 716 were cases with HIV infections and 791 were controls without infection. The ACASI were conducted from April 2009 to March 2017.

Table 1 presents the sociodemographic characteristics of all study participants. Female donors compared to males were less likely to be HIV positive (OR: 0.62; 95 % CI: 0.49–0.78). Donors from Fundação Pró-Sangue (OR: 0.58; 95 % CI: 0.43–0.76), Hemominas (OR: 0.38; 95 % CI: 0.28–0.51) and Hemorio (OR: 0.71; 95 % CI: 0.54–0.92) showed lower associations with HIV infection when compared to donors from Hemope. According to the educational level, people with incomplete high school educations were more likely to be HIV positive when compared to individuals with higher levels of education (OR: 1.30; 95 % CI: 1.04–1.63). Donors who reported being separated, divorced or widowed (OR: 4.28; 95 % CI: 2.81–6.59), followed by the single and never married group (OR: 3.55; 95 % CI: 2.58–4.91) and people who live together without being married (OR: 3.62; 95 % CI: 2.80–4.72) were more likely to be HIV positive. Donors defined as bisexual (OR: 27.14; 95 % CI: 4.02–60.90) and homosexual (OR: 8.33; 95 % CI: 5.63–12.69) presented higher ORs when compared to heterosexuals.

**Table 1:** Sociodemographic characteristics of donors according to HIV status among Brazilian blood donors

Sociodemographic characteristic	Control	Case	OR (95 % CI)	P-value
	(*n* = 791)	(*n* = 716)		
**Gender**				
Male	556 (70.3)	568 (79.3)	1.00	–
Female	235 (29.7)	148 (20.7)	0.62 (0.49; 0.78)	**<0.001**
**Age** (*n* = 1506)				
18–25	189 (23.9)	176 (24.6)	1.00	–
26–30	130 (16.4)	150 (21.0)	1.24 (0.91; 1.69)	0.178
31–39	235 (29.7)	205 (28.7)	0.94 (0.71; 1.24)	0.645
40+	237 (30.0)	184 (25.7)	0.83 (0.63; 1.10)	0.205
**Blood Donation Center**				
Hemope	194 (24.5)	264 (36.9)	1.00	–
Fundação Pró-Sangue	194 (24.5)	152 (21.2)	0.58 (0.43; 0.76)	**<0.001**
Hemominas	195 (24.7)	100 (14.0)	0.38 (0.28; 0.51)	**<0.001**
Hemorio	208 (26.3)	200 (27.9)	0.71 (0.54; 0.92)	**0.011**
**Donation type**				
Community	362 (58.6)	371 (61.9)	1.00	–
Replacement	256 (41.4)	228 (38.1)	0.87 (0.69; 1.09)	0.231
**Donor type** (*n* = 1261)				
First time	311 (48.7)	285 (45.8)	1.00	–
Repeat	328 (51.3)	337 (54.2)	1.12 (0.90; 1.40)	0.311
**Education** (*n* = 1502)				
< High School	206 (26.1)	225 (31.5)	1.30 (1.04; 1.63)	**0.022**
≥ High School	582 (73.9)	489 (68.5)	1.00	–
**Marital status** (*n* = 1506)				
Married	326 (41.3)	115 (16.1)	1.00	
Single, never married	298 (37.7)	381 (53.2)	3.62 (2.80; 4.72)	**<0.001**
Living together, never married	119 (15.1)	149 (20.8)	3.55 (2.58; 4.91)	**<0.001**
Separated/Divorced	47 (5.9)	72 (9.9)	4.28 (2.81; 6.59)	**<0.001**
Widowed				
**Sexual orientation** (*n* = 1447)				
Heterosexual	713 (94.8)	417 (60.0)	1.00	–
Bisexual	8 (1.1)	127 (18.3)	27.14 (14.02; 60.90)	**<0.001**
Homosexual	31 (4.1)	151 (21.7)	8.33 (5.63; 12.69)	**<0.001**

OR, odds ratio; 95 % CI, 95 % confidence interval.

Table 2 shows the association between risk behaviors according to HIV status, such as workplace exposure, piercing, tattooing, blood transfusion, intravenous drug use (IVDU), acupuncture, hair removal, among others. Individuals who had already used intravenous drugs at least once in their lives or had IVDU sex partners had a greater association with HIV infection (OR: 6.77; 95 % CI: 3.58–14.17). inmate populations (OR: 3.19; 95 % CI: 1.32–8.90) or individuals who have had sexual partners who were inmates at least once in their lives (OR: 4.35; 95 % CI: 2.42–8.42) were more likely to be HIV positive. Being MSM (OR: 23.54; 95 % CI: 15.90–36.12), having MSM sex without condom use (OR: 21.64; 95 % CI: 13.95–35.23) and sexual partners of MSM (OR: 20.02; 95 % CI: 13.92–29.72) were associated with HIV infection. Likewise, individuals who had sex with an HIV positive partner (OR: 23.38; 95 % CI: 11.67–55.58) were more likely to be HIV positive. However, many of the univariate data presented in Table 2 lost significance in Table 3, which presents multivariate data.

**Table 2:** Risk behaviors according to HIV status among Brazilian blood donors

Characteristic	Control	Case	OR (95 % CI)	P-value
	(*n* = 791)	(*n* = 716)		
Potential job exposure, ever (*n* = 1503)	33 (4.2)	104 (14.6)	3.92 (2.65–5.98)	**<0.001**
Piercing, ever (*n* = 1504)	96 (12.1)	159 (22.3)	2.08 (1.58–2.75)	**<0.001**
Tattoo, ever (*n* = 1504)	106 (13.4)	178 (25.0)	2.15 (1.65–2.81)	**<0.001**
Acupuncture, ever (*n* = 1503)	46 (5.8)	47 (6.6)	1.14 (0.75–1.74)	0.537
Manicure or Shave in salon/barber shop, ever (*n* = 1504)	371 (46.9)	436 (61.2)	1.78 (1.45–2.19)	**<0.001**
Medical procedures in the last 12 months (*n* = 966)	152 (31.9)	251 (51.2)	2.24 (1.73–2.91)	**<0.001**
Surgery in the last 12 months (*n* = 941)	126 (27.3)	221 (46.1)	2.28 (1.74–3.00)	**<0.001**
Endoscopy in the last 12 months (*n* = 296)	40 (27.4)	68 (45.3)	2.20 (1.36–3.59)	**0.001**
IVDU or sexual partner of IVDU, ever (*n* = 1506)	10 (1.3)	57 (8.0)	6.77 (3.58–14.17)	**<0.001**
Blood Transfusion/Sex partner with blood transfusion, ever (*n* = 1506)	42 (5.3)	62 (8.7)	1.69 (1.13–2.56)	**0.011**
Inmate, ever (*n* = 1504)	6 (0.8)	17 (2.4)	3.19 (1.32–8.90)	**0.015**
Sex with inmate, ever (*n* = 1474)	13 (1.7)	50 (7.0)	4.35 (2.42–8.42)	**<0.001**
MSM (*n* = 1123)	29 (5.2)	320 (56.4)	23.54 (15.90–36.12)	**<0.001**
MSM, unprotected sex last 12 months (*n* = 1051)	22 (4.0)	239 (47.5)	21.64 (13.95–35.23)	**<0.001**
Sexual partner of MSM, ever (*n* = 1489)	33 (4.3)	337 (47.1)	20.02 (13.92–29.72)	**<0.001**
Sex with person with potential job exposure, ever (*n* = 1474)	41 (5.4)	80 (11.2)	2.23 (1.51–3.32)	**<0.001**
Sex with HIV+ person, ever (*n* = 1475)	7 (0.9)	127 (17.8)	23.38 (11.67–55.58)	**<0.001**
2 or more heterosexual partners, protected sex, last 12 months (*n* = 1452)	27 (3.6)	16 (2.3)	0.62 (0.32–1.15)	0.136
2 or more heterosexual partners, last 12 months (*n* = 1440)	127 (17.0)	153 (22.1)	1.39 (1.07–1.81)	**0.013**
Sex with sex worker, protected sex, last 12 months (*n* = 1388)	1 (0.1)	3 (0.4)	3.14 (0.40–63.61)	0.322
Sex with unknown person, protected sex, last 12 months (*n* = 1369)	7 (1.0)	11 (1.6)	1.65 (0.65–4.51)	0.303
Sex with sex worker/unknown or ≥2 heterosexual partners, unprotected sex, last 12 months (*n* = 1469)	31 (4.1)	27 (3.8)	3.14 (0.40–63.61)	0.322

OR, odds ratio; 95 % CI, 95 % confidence interval; IVDU, intravenous drug use; MSM, men who have sex with men.

**Table 3:** Multivariate analysis of factors associated with HIV infection among Brazilian blood donors

Characteristic	OR	95 % CI	P-value
**BOTH GENDERS**			
**Education**			
≥ High School	1.00	–	–
< High School	2.91	(1.93–4.42)	**<0.001**
**Marital status**			
Married	1.00	–	–
Living together, never married	3.20	(1.90–5.40)	**<0.001**
Single, never married	3.47	(2.23–5.48)	**<0.001**
Separated/Divorced	3.97	(2.26–8.47)	**<0.001**
Widowed			
**Sexual orientation**			
Heterosexual	1.00	–	–
Homosexual	2.45	(1.07–5.73)	0.035
Bisexual	7.55	(2.60–27.60)	**<0.001**
**Behavioral factors (ref. No)**			
Manicure or Shave in salon/barber shop, ever	1.71	(1.17–2.50)	**0.005**
Tattoo, ever	1.86	(1.18–2.93)	**0.008**
Medical procedures in the last 12 months	2.60	(1.81–3.76)	**<0.001**
2 or more heterosexual partners, last 12 months	2.64	(1.74–4.05)	**<0.001**
Potential job exposure, ever	3.14	(1.69–5.92)	**<0.001**
IVDU or sexual partner of IVDU, ever	5.75	(1.88–21.89)	**0.004**
Sexual partner of MSM, ever	11.78	(6.17–23.53)	**<0.001**
Sex with HIV+ person, ever	15.57	(5.78–54.62)	**<0.001**
			
**MALE**			
**Sexual orientation**			
Heterosexual	1.00	–	–
Homosexual	5.71	(2.13–17.13)	**<0.001**
Bisexual	8.10	(2.47–36.66)	**0.002**
**Behavioral factors (ref. No)**			
Manicure or Shave in salon/barber shop, ever	1.65	(1.10–2.49)	**0.016**
Medical procedures in the last 12 months	1.78	(1.18–2.70)	**0.006**
Tattoo, ever	2.03	(1.18–3.52)	**0.011**
2 or more heterosexual partners, last 12 months	2.33	(1.48–3.70)	**<0.001**
Potential job exposure, ever	2.81	(1.44–5.60)	**0.003**
MSM	8.30	(4.18–17.37)	**<0.001**
IVDU or sexual partner of IVDU, ever	8.31	(2.19–54.71)	**0.007**
Sex with HIV+ person, ever	5.75	(1.69–26.73)	**0.010**
			
**FEMALE**			
**Sexual orientation**			
Heterosexual	1.00	–	–
Homosexual	0.07	(0.01–1.01)	0.094
Bisexual	14.35	(1.89–297.81)	**0.023**
**Behavioral factors (ref. No)**			
Tattoo, ever	2.72	(1.32–5.73)	**0.007**
Medical procedures in the last 12 months	4.11	(2.15–8.12)	**<0.001**
Sexual partner of MSM, ever	5.06	(1.17–27.31)	**0.039**
2 or more heterosexual partners, last 12 months	6.38	(2.66–16.71)	**<0.001**
Sex with HIV+ person, ever	88.60	(15.58–1924.91)	**<0.001**

OR, odds ratio; 95 % CI, 95 % confidence interval; IVDU, intravenous drug use; MSM, men who have sex with men.

Adjusted ORs are presented for the risk factors associated with HIV infection from the multivariable analysis in Table 3. Characteristics that were independently associated with HIV infection were lower level of education (OR 2.93; 95 % CI: 1.93–4.42), donors who reported being separated, divorced or widowed, (OR: 3.97; 95 % CI: 2.26–8.47), followed by the single and never married group (OR: 3.47; 95 % CI: 2.23–5.48) and people who live together without being married (OR: 3.20; 95 % CI: 1.90–5.40). Behavioral factors such as workplace exposure (OR: 3.14; 95 % CI: 1.69–5.92), tattooing (OR: 1.86; 95 % CI: 1.18–2.93), manicuring or shaving in beauty salons or barber shops (OR: 1.71; 95 % CI: 1.17–2.50), medical procedures within the last 12 months (OR: 2.60; 95 % CI: 1.81–3.76), IVDU or IVDU partner (OR: 5.75; 95 % CI: 1.88–21.89), MSM sexual partner (OR: 11.78; 95 % CI: 6.17–23.53), sex with an HIV positive partner (OR: 15,57; 95 % CI: 5.78–54.62), having had two or more heterosexual partners within the last 12 months (OR: 2.64; 95 % CI: 1.74–4.05) remained associated with greater odds of being HIV positive. Table 3 also shows the multivariate analysis stratified by gender. Male blood donors who were classified as bisexual (OR: 8.10; 95 % CI: 2.47–36.66) or homosexual (OR: 5.71; 95 % CI: 2.13–17.13) were more likely to be HIV positive. The analyzed behaviors, such as exposure at work (OR: 2.81; 95 % CI: 1.44–5.60), tattooing (OR: 2.03; 95 % CI: 1.18–3.52), manicuring or shaving in beauty salons or barber shops (OR: 1.65; 95 % CI: 1.10–2.49), medical procedures within the last 12 months (OR: 1.78; 95 % CI: 1.18–2.70), IVDU or partner IVDU (OR: 8.31; 95 % CI: 2.19–54.71), being MSM (OR: 8.30; 95 % CI: 4.18–17.37), sex with an HIV positive partner (OR: 5.75; 95 % CI: 1.69–26.73), having had two or more heterosexual partners within the last 12 months (OR: 2.33; 95 % CI: 1.48–3.70) when reported by male donors were more associated with being HIV positive. On the other hand, female blood donors were more likely to be HIV positive when classified in the bisexual group (OR: 14.35; 95 % CI: 1.89–297.81), and when they reported behaviors such as tattooing (OR: 2.72; 95 % CI: 1.32–5.73) medical procedures within the last 12 months (OR: 4.11; 95 % CI: 2.15–8.12), a MSM sexual partner (OR: 5.06; 95 % CI: 1.17–27.31), sex with an HIV positive partner (OR: 88.6; 95 % CI: 15.58–1924.91) and having had two or more heterosexual partners within the last 12 months (OR: 6.38; 95 % CI: 2.66–16.71).

## Discussion

This study shows that male donors are still more likely to be HIV positive, as demonstrated in other researches published in the same period,^21^ probably because the epidemiology of the states in the present study is predominantly clade B, which means a greater prevalence of sexual infection. The greater chance of being HIV positive found among blood donors from Recife probably is also related to the number of partners and other regional issues, such as disparity in the availability of resources, such as reduced access to prevention and health promotion. At the time of this study the number of sexual partners allowed for donor candidates was different at study sites: Hemominas, in Minas Gerais, allowed up to two partners, while Fundação Pró-Sangue in Sao Paulo and Hemope in Pernambuco allowed up to three partners. Previous studies have shown that there were differences in the deferral criteria between blood centers and this can have had a direct impact on the chances of having an HIV positive donor.^17^^,^^22^

Low education was also a factor associated with greater chances of being HIV positive. Access to information and mass education are fundamental components in strategies to reduce the incidence and prevalence of HIV in populations. However, the interaction between sociocultural, political and economic issues and its relation with social and sexual behavior cannot be underestimated. The diversity of socio-epidemiological risk exposure interferes in this process, differentiating individuals in terms of their knowledge about HIV and their behavior.^23^, ^24^, ^25^ In the current sample, marital status was shown to be a protective factor when compared to unmarried donors, as in other studies.^18^^,^^26^ Theoretical epidemiological models on HIV/AIDS transmission ignore marital status as a risk factor.^27^ Marriage is considered an aggravating or protective risk of infection between people, with contradictory results in different studies.^28^^,^^29^ According to a social psychology study, there is a higher prevalence of HIV in MSM associated with a context of exclusion and discrimination of these individuals, which impacts access to health care.^30^ When experiencing prejudice in public health services, for example, MSM and transwomen may begin to avoid such services, due to the expectation of discrimination.^31^ The CDC performed a study^32^ emphasizing the importance of annual testing for the MSM population and the importance of prevention programs to improve their strategies to reach the population at greatest risk, aiming to facilitate this population's access to testing and treatment services and, consequently, to provide more information and care thereby reducing the spread of HIV. When analyzing risk behaviors, the rate of infection through IVDU is probably related to the frequency of use, the sharing of needles and poor access to effective HIV control and prevention therapies.^33^

An unexpected finding was the greater chance of being HIV positive in donors with the habit of going for manicures or shaves in beauty salons or barber shops. There are several studies that support the risk of other blood-borne infections, such as hepatitis and syphilis, through unsafe tattooing practices,^34^, ^35^, ^36^ although the evidence is less clear when it comes to HIV transmission.^36^^,^^37^ Although this transmission pattern of HIV is not common or widely reported, a few studies report this pattern of increased risk for HIV transmission.^38^ It is likely that there are confounding factors involved in these results, probably behavioral factors. More studies are needed to highlight the lack of knowledge on the factors involved in this pattern of HIV infection in people who perform these practices. The association of HIV in people who undergo aesthetic procedures may be associated with the popularity of these procedures, especially for young people. The risk in the prison population is also associated with HIV infection in the present study. These factors deserve more attention.^34^

As to increased risk sexual behaviors, some widely reported in the literature are heterosexual sex with two or more partners within the last 12 months, being MSM, unprotected sex with an MSM partner, as well as sex with an HIV-positive partner. The latter three have the highest ORs found in the univariate and multivariate analysis for both males and females in the present study.^15^, ^16^, ^17^, ^18^^,^^39^^,^^40^ When the behavioral factors associated with HIV infection between men and women are compared, being bisexual or having MSM sexual partners are increased risk factors for both, with the magnitude of the ORs being greater in women.^41^

Almeida-Neto et al.^8^ reported on HIV risks in blood donors in Brazil with similar data, but their donors were recruited between 2009 and 2011. The present study evaluated data from donors recruited from 2009 to 2017. The current results do not differ substantially from previous data, showing a tendency towards the maintenance of increased risk behaviors already identified, but this study shows a significant relationship between HIV and women who reported being bisexual, which was not reported in the previous publication.

This study has limitations. One limitation is that only factors in the ACASI interview were evaluated. Therefore, it is possible that other risk factors could be attributed to greater exposure to HIV and were not identified in the present study, such as questions about the use of pre- and post-exposure prophylaxis, having had some sexually transmitted diseases, such as chlamydia or gonorrhea, or Early age at first intercourse. Another limitation is that the study was conducted from 2009 to 2017, and the profile of donors may have changed since then. Furthermore, it was a period prior to the recent change in the screening criteria of Brazilian blood centers that occurred in 2020, which censored questions about the sexual orientation of donors in clinical screening.^42^ These changes may have an impact on the profile of donors and further studies are needed to understand the new profile of donors after this change.

## Conclusion

After analyzing the findings of the present study, there are factors that lead to an increased risk of acquiring HIV. Many have already been widely reported in the literature, but persist over time, such as IVDU, behaviors related to sexual activity, such as being bisexual (for men and women) and multiple sexual partners. More studies are needed to better understand these increased risk factors in order to develop policies to approach the most vulnerable populations.

This study shows that the donor profile has not changed over the years, there is little variability related to risk factors. In 2019 there were specific changes in the screening criteria in Brazilian blood centers, such as the removal of restrictions on blood donations by MSM. This fact, associated with the use of pre- and post-exposure antiretroviral medications, can generate significant changes. Therefore, this pattern must be constantly monitored by new studies, especially due to the high transmissibility rate of the virus.

## Conflicts of interest

None.
